# Electronic Structure and Mesoscopic Simulations of Nonylphenol Ethoxylate Surfactants. A Combined DFT and DPD Study

**DOI:** 10.3390/molecules18089441

**Published:** 2013-08-07

**Authors:** Diego Valencia, Jorge Aburto, Isidoro García-Cruz

**Affiliations:** 1 Programa de Ingeniería Molecular, Instituto Mexicano del Petróleo, Eje Central Lázaro Cárdenas 152, Col. San Bartolo Atepehuacan, C.P. 07730, Mexico City, Mexico; 2 Programa de Procesos de Transformación, Instituto Mexicano del Petróleo, Eje Central Lázaro Cárdenas 152, Col. San Bartolo Atepehuacan, C.P. 07730, Mexico City, Mexico; E-Mail: jaburto@imp.mx

**Keywords:** nonylphenol ethoxylate, surfactant, DFT, DPD, micelles, solvation energy

## Abstract

The aim of this work was to gain insight into the effect of ethylene oxide (EO) chains on the properties of a series of nonylphenol ethoxylate (NPE) surfactants. We performed a theoretical study of NPE surfactants by means of density functional theory (DFT) and dissipative particle dynamics (DPD). Both approximations were used separately to obtain different properties. Four NPEs were selected for this purpose (EO = 4, 7, 11 and 15 length chains). DFT methods provided some electronic properties that are related to the EO units. One of them is the solvation Gibbs energy, which exhibited a linear trend with EO chain length. DPD calculations allow us to observe the dynamic behavior in water of the NPE surfactants. We propose a coarse-grained model which properly simulates the mesophases of each surfactant. This model can be used in other NPEs applications.

## 1. Introduction

Surface active agents (surfactants) compounds have been used for several applications [1]. The surfactant structure determines most of their properties and behavior. In general, surfactants are formed by polar and non-polar groups, which can or not have a charge. Surfactant-solvent interaction involves molecular recognition of the accurate groups in the surfactant molecule. As a consequence of these interactions the surfactant exhibited a series of mesophases such as micelles, hexagonal, cubic and lamellar structures, with different geometric forms [2]. These mesophases are called lyotropic liquid crystals because the transition between them is driven by the surfactant-solvent interactions. These interactions depend on concentration and temperature.

Non-ionic surfactants bear no apparent ionic charge. Non-ionic surfactants containing ethylene oxide (EO) chains have found widespread use as emulsifiers in the agricultural, paint and cosmetics industries [3]. Their molecular structure contains repeated EO units as hydrophilic groups. Surfactant properties depend on EO length and type of hydrophobic combined groups. Nonylphenol ethoxylate (NPE) surfactants have been used in EO range from 4 to 70 units. The critical micelle concentration (CMC), the concentration where the surfactant monomers start the aggregation to form micelles, of NPE varies depending on the number of EO units [4]. It has been shown the more EO units, the higher the CMC values. This indicates that aggregation is less favorable when the number of hydrophilic groups increases.

Recently, the properties of surfactants have been exploited in enhanced oil recovery (EOR) for the petroleum industry [5]. There is an obvious motivation to develop this technique due to the fact that there is a growing interest in the use of non-conventional heavy and extra-heavy oil resources to produce fuels and petrochemicals all around the World [6]. The incorporation of heavy oil into the energy market presents important challenges that require significant technological developments in the production chain. The transportation of heavy and extra-heavy oil presents many operational difficulties that limit their economic viability. In this context, EOR optimization should profit from the systematic use of molecular modeling tools developed and optimized during the last decades [7].

Surfactant characteristics have been understood to select the specific molecule for a specific application. In this line, computational chemistry methods have been used to simulate surfactants to get a deeper understanding of their atomistic and electronic properties [8]. Furthermore, surfactant properties have been studied by several approximations. Methods used in computational chemistry vary depending on the studied system. One of them is the DFT method which has provided information about the electronic structure of certain surfactants due to the presence of functional groups. Some properties of DFT calculations are attractive to understand the surfactant behavior. Based on DFT calculations it is possible to obtain the solvation energy of dispersants [9]. This energy is an important feature of each surfactant and it is related to the molecule structure and the polarity of the solvent used. There are some approximations to treat the solvent effects. In one of them, the solvent is treated as a dielectric medium continuum, usually characterized by a bulk dielectric constant.

DFT methods provide several properties with accuracy, but this methodology is unable to understand the dynamic behavior of the surfactants. Those types of studies can be performed using dissipative particle dynamics (DPD) [10], a methodology that combines molecular dynamics with coarse-grained Brownian dynamics. DPD allows simulations on mesoscopic space and time scales, significantly exceeding the limits of molecular dynamic simulations while accurately addressing hydrodynamic interactions. For instance, some works have been performed for polymer-based surfactants with proper description of physical properties [11,12,13].

In this work, we studied NPE surfactants with 4, 7, 11 and 15 EO units. This work tries to understand the behavior of these systems in aqueous media and provide a proper description for them to possible use in EOR processes. To accomplish this purpose, two approximations have been used: (i) electronic structure with all electron DFT and (ii) coarse-grained DPD calculations. 

## 2. Results and Discussion

### 2.1. DFT Calculations

**Figure 1:**
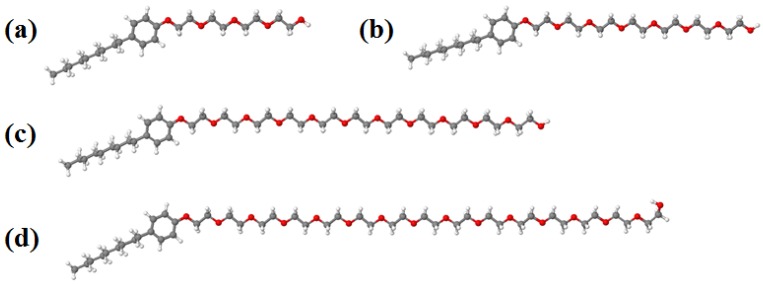
Optimized geometries for: (**a**) NPE-4, (**b**) NPE-7, (**c**) NPE-11 and (**d**) NPE-15 surfactants. Color atoms: ● O; ● C and ○ H.

The optimized structures of NPEs are shown in Figure 1. The surfactants vary in the EO units in the hydrophilic region. It is clearly observed that EO units exhibit a linear chain for all the studied compounds. Optimized geometries of NPEs surfactants represent the ground state of the molecules, as we can see the *sp^3^* hybridization of the atoms in EO and C_9_ chains results in a linear structure. However, their geometries could be modified when the molecules interact with solvents due to different and new interactions will be created between them (e.g., hydrophilic and hydrophobic ones). In that case a nonlinear structure of the EO and C_9_ chains which can be studied in molecular dynamics simulations can be expected.

Energy values of the HOMO and LUMO orbitals (frontier orbitals) are almost the same for all the surfactants (Table 1), E_HOMO_ is around −5.98 eV and E_LUMO_ −0.38 eV. The ΔE_H-L_ gap shows a small increase from 5.58 eV (NPE-4) to 5.62 eV (NPE-15) as the EO units increase. Furthermore, there is no effect on both molecular orbitals due to the EO units. This makes sense because they are not electron delocalized molecules. We analyzed the oxygen atom charges of the NPE surfactants. We note two main differences in the O charges; the O atom from the OH has a larger (−0.495) value than the O atoms from EO (−0.292). The chemical environment of both O atoms is different; one of them is a CH_2_-O-CH_2_ and the other CH_2_-O-H, respectively. Those differences could have implications in the aggregation process due to that fact there are not the equal O charges in the same molecule. The electronic structure of different O atoms in the same molecule can result in different modes of interaction with the solvent. Furthermore, the H bonding between NPEs and water can produce changes in conformational geometries and their aggregation behavior.

**Table 1:** Electronic properties, charge of O atoms and solvation Gibbs energy in water of NPE surfactants at B3LYP/6-31++G(d,p) level.

Surfactant	E_HOMO_ (eV)	E_LUMO_ (eV)	ΔE_H-L_ (eV)	*q*O (EO)	*q*O (OH)	ΔG_solv_ (kcal/mol)
NPE-4	−5.97	−0.39	5.58	−0.286	−0.495	−19.13
NPE-7	−5.98	−0.38	5.60	−0.295	−0.495	−25.09
NPE-11	−5.98	−0.38	5.60	−0.292	−0.495	−33.00
NPE-15	−5.98	−0.37	5.62	−0.292	−0.495	−40.25

**Figure 2:**
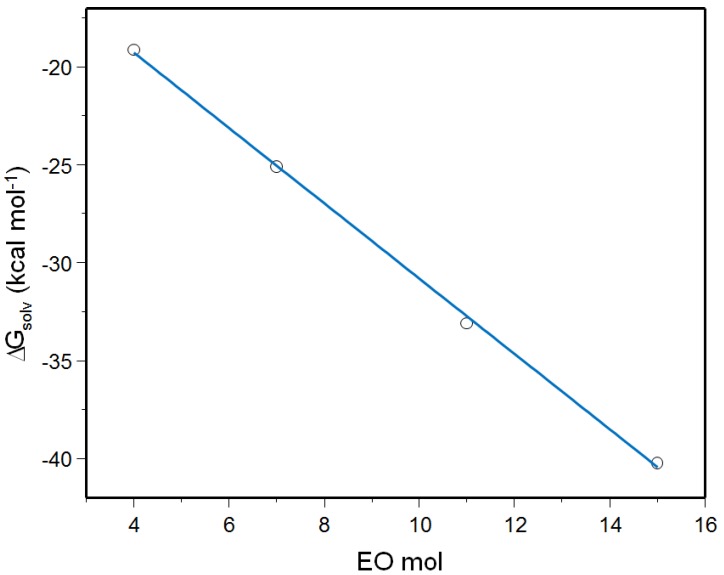
Relationship between ΔG_solv_ and EO units of NPE surfactants.

The solvation Gibbs energy (ΔG_solv_) was calculated for the surfactants in water using the conductor-like polarizable continuum model (CPCM) model, which is based on the polarized continuum model (PCM). In this method, the solute cavities are modeled based on the optimized molecular shape, and include both electrostatic and nonelectrostatic contributions to the energies [14]. ΔG_solv_ of each molecule is important to understand the behavior of NPEs in aqueous solution. The values of this process are shown in Table 1. We also plotted the ΔG_solv_ values *vs*. the EO units in Figure 2 and obtained the equation of the corresponding linear regression [Equation (1)]. The relationship between both values exhibited a linear plot. The coefficient of determination of Equation (1) is 0.9993, pointing out the good linearity. Based on Equation (1) it is possible to obtain the ΔG_solv_ for other NPE surfactants which were not studied in this work. 

(1) $$\Delta G_{solv}=-1.92\left(EO\right)-11.56$$

The results are in good agreement with the CMC values of each surfactant. The ΔG_solv_ is related to the interaction of surfactant (as a monomer) with a solvent, in this case water. On the other hand, CMC represents the concentration when the surfactant starts to form micelles. For instance, ΔG_solv_= −40.25 kcalmol^−1^ and CMC = 90 ppm for NPE-15 and ΔG_solv_= −25.09 kcalmol^−1^ and CMC = 39 ppm for NPE-7 [15]. It can be seen the interaction of NPE-15 with the solvent is more favorable than the one for NPE-7. It means that the NPE-15 monomer has the larger interaction with water and, as a consequence, the aggregation process occurs at higher concentration.

### 2.2. DPD Simulation

**Figure 3:**
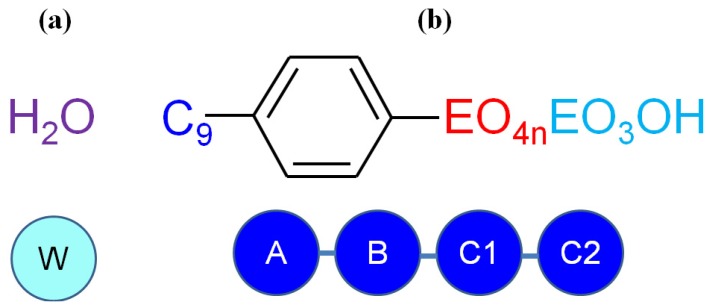
Molecules used in DPD simulations and their respective coarse-grained model (**a**) water (solvent), (**b**) NPE (surfactant), where the EO chain can be seen as: NPE-4 (EO_4_OH); NPE-7 (n = 1), NPE-11 (n = 2), NPE-15 (n = 3).

The coarse-grained models of the molecules used in the DPD calculations are shown in Figure 3. Water, nonyl, benzene, EO_4_ and EO_3_OH groups were represented by DPD beads: W, A, B, C1 and C2, respectively. We consider this partition is proper to represent the NPEs dynamic behavior in water because we selected the distinct chemical characteristics in each bead. We also performed DPD calculations with EO units as a coarse-grained model, but this representation failed to show the morphologies of the mesophases (not shown).

At the beginning of DPD calculations it is necessary to set the interaction parameters between all the beads. Table 2 gathers the values of all the interaction parameters used in this study. The values were calculated with Equations (3) and (4) (see below).

**Table 2:** Interaction parameters *a_ij_* used in these simulations.

	W	A	B	C1	C2
W	25.00	55.01	34.89	47.15	39.44
A	55.01	25.00	34.35	30.65	32.40
B	34.89	34.35	25.00	29.93	25.62
C1	47.15	30.65	29.93	25.00	27.03
C2	39.44	32.40	25.62	27.03	25.00

**Figure 4:**
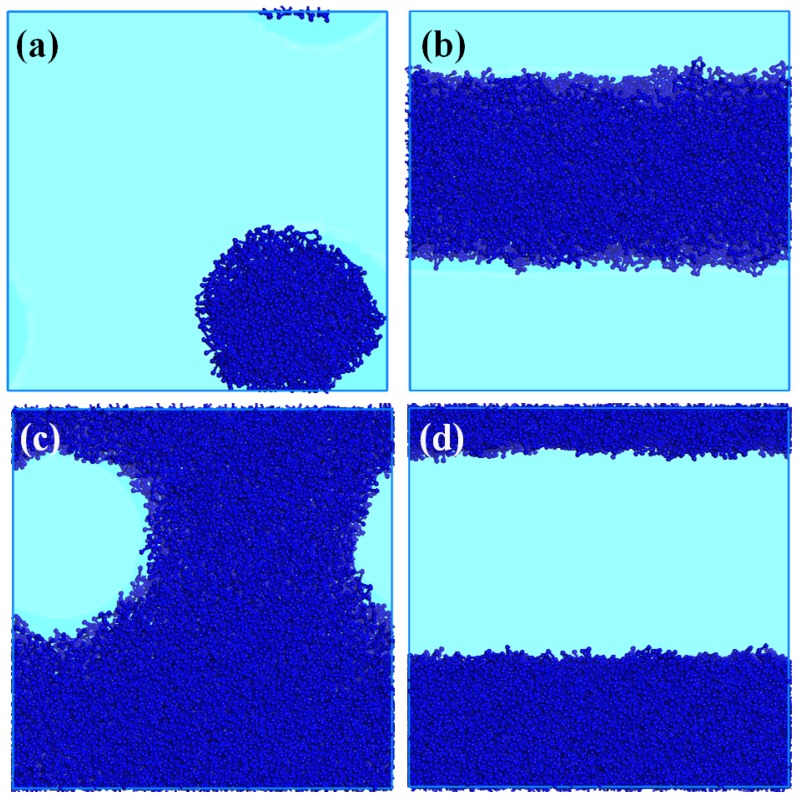
Phase regions with the DPD calculated phases at T = 298.15 K and water: NPE-15 ratio: (**a**) 1:0.05, (**b**) 1:0.25, (**c**) 1:0.5 and (**d**) 1:0.75. In all the Figures, ball and sticks represent the surfactant molecules.

As we mentioned above, the concentration of surfactant in water has consequences for the phase morphology. DPD simulations have been used to describe this effect. The correct phase behavior of the NPE surfactants should be reproduced by the simulations. In Figure 4, it can be seen that spherical micelles were formed at lower concentrations. Then, as the concentration is increased, the mesophases are transformed into rod-like, cubic and lamellar morphologies. The water is omitted in all figures for clarity. The hydrophobic (A and B) and hydrophilic beads (C1 and C2) are pointing outside as well as inside. We obtained the mesophases for the others surfactants in this study (not shown). Therefore, it is clearly that these coarse-grained models used to represent the NPEs are proper to simulate the dynamic behavior of these molecules. The models can be used in more complex systems such as EOR process, where there is larger number of species and interactions between them.

Based on our DPD results, we calculated the average end-to-end distance, which is a physical property of polymers. The end-to-end distance of a linear polymer chain averages over all conformations of the chain [16]. Therefore, the end-to-end distance of the NPEs can provide useful information on their aggregation process. The end-to-end distances for the NPEs studied are shown in Table 3 for a set of different concentration in water. The end-to-end distance is in good agreement with the EO units in the surfactant molecule. At a fixed concentration the end-to-end distance has the trend: NPE-15 > NPE-11 > NPE-7 > NPE-4. For instance, NPE-15 (2.06), NPE-11 (1.86), NPE-7 (1.61) and NPE-4 (1.32) at 0.01 DPD units. It can be seen this trend for the concentration range studied, which is clearly related to the EO chain size. Furthermore, these values pointing out the effect of EO units on the aggregation size of these NPEs. Another feature of the end-to-end distance is a constant value between 0.05 and 0.1 concentration. It is important to note that this constant value can be related to the mesophases formation due to the polymer agglomeration in water.

**Table 3:** Average end-to-end distance of NPE surfactants.

Surfactant	Surfactant concentration (DPD units)							
	0.001	0.005	0.01	0.05	0.10	0.25	0.5	0.75
NPE-4	0.99	1.29	1.32	1.36	1.37	1.37	1.38	1.38
NPE-7	1.43	1.59	1.61	1.66	1.67	1.68	1.69	1.70
NPE-11	1.62	1.81	1.86	1.91	1.93	1.94	1.95	1.96
NPE-15	1.84	2.01	2.06	2.14	2.15	2.17	2.18	2.19

As we mentioned above, the properties calculated by means of DFT methods provide information about the electronic structure of NPE surfactants which can be analyzed in the ground state. However, the dynamic behavior of these surfactants was studied by DPD simulations. In this line, both approximations can complement each other. DFT calculations allow us to understand the linear geometry of EO chains for the ground state, the O charges and the ΔG_solv_ of NPEs molecules. We noted that O charges in the surfactant molecules exhibited differences because of the non-equivalence of the O atoms in the molecule. The agglomeration of NPEs in aqueous solutions as we see in the DPD simulations may be explained for the distinct O atoms which can interact with water in different ways. On the other hand, DPD simulations allow us to set up a proper manner to represent the NPEs by these coarse-grained models. Further studies could use this representation to evaluate the behavior of NPEs for other fields such as EOR process.

## 3. Computational Details

We performed all electron DFT calculations of a series of NPEs compounds. Those are NPE-4, NPE-7, NPE-11 and NPE-15. The geometries for all compounds were fully optimized. We used the well-known hybrid B3LYP [17,18] functional with the 6-31++G(d,p) basis set. Solvation energies were computed at the same level of theory for each surfactant in water with the conductor-like polarizable continuum model (CPCM) [14]. In this approach, the cavity of the surface is partitioned into small domains called tesserae in such a way that the molecular shape is represented by interlocking spheres centered at the solute atoms or functional groups. All the calculations were carried out in the Gaussian 03 software [19].

On the other hand, the dynamic behavior of the surfactants in water was studied by dissipative particle dynamics (DPD) approximation. In the DPD method, the time evolution of the interacting particles is governed by Newton’s equation of motion. Dimensionless units are used in DPD simulations and the mass of each bead is usually set to 1 DPD mass unit [20]. In the DPD model, the total force of beads is given in Equation (2):

(2) $$F_{i}=\underset{i\neq j}{\sum }F_{ij}^{C}+\underset{i\neq j}{\sum }F_{ij}^{D}+\underset{i\neq j}{\sum }F_{ij}^{R}$$

where the conservative force $F_{ij}^{C}$ is a soft repulsive force, the dissipative force $F_{ij}^{D}$ is a frictional force and the random force $F_{ij}^{R}$ is a random interaction between a bead *i* and *j*.

In the simulation, the radius of interaction, the particle mass, and the temperature were chosen as *r_c_* = *m* = *k_B_T* = 1 and *σ* = 3.67, while the particle density *ρ* = 3. The only parameter to be determined is the maximum repulsive force $a_{ij}$, which is chosen according to the linear relation with Flory-Huggins $\chi_{ij}$ parameters. The parameter $a_{ij}$ depends on the underlying atomistic interactions and is related to the Flory–Huggins through [21]:

(3) $$a_{ij}=a_{ii}+3.26\chi_{ij}$$

In this way, there is a connection between the molecular character of the coarse-grained model and the DPD parameter. The parameter $a_{ij}$ is given in terms of *k_B_T* (DPD reduced units). Using Equation (3) implies that if the species are compatible, $\chi_{ij}\approx 0$, and therefore,$\;a_{ij}=25$. On the other hand, the $\chi_{ij}$ parameter between DPD pairs of particles can be obtained from the calculation of the mixing energy [22] (Equation 4) with Blends module:

(4) $$\chi_{ij}=\frac{Z_{12}E_{12}+Z_{21}E_{21}-Z_{11}E_{11}+Z_{22}E_{22}}{2RT}$$

Surfactant dynamic behavior was simulated using periodic boundary condition cells of 30 × 30 × 30 DPD length units. The initial configuration of the boxes was constructed with a random distribution of the molecules. All the simulations were carried out considering a NVT ensemble with 10^6^ steps, with Δt = 0.05 DPD time units. The calculations were carried out in the DPD software available in the Materials Studio package [23].

## 4. Conclusions

We have described the effect of EO units in NPEs surfactants by theoretical calculations based on two distinct approximations. Both are complementary and allow us to explain the physical properties of NPEs and their interaction with water. Electronic properties were obtained by means of DFT calculations. We observed a negligible effect of the number of EO units in the frontier orbitals energy. However, O atoms charges in the NPEs are not equivalent and they can interact with water in different ways. The solvation Gibbs energy showed a linear trend with respect to the number of EO units, and a linear equation was obtained. This property explains the different interaction energy with the solvent (water) of the NPEs due to the EO units. On the other hand, the dynamic behavior of these surfactants was studied by DPD simulations. Coarse-grained models proposed in this study are proper representations of NPEs, and the mesophases were detected in a concentration range. Moreover, the end-to-end distances of NPEs are in good agreement with the NPEs size imposed by the EO units. We consider these coarse-grained models can be useful for further NPEs applications which need molecular dynamics simulations.

## References

[1] Schramm L.L., Stasiuk E.N., Marangoni D.G. (2003). Surfactants and their applications. Annu. Rep. Prog. Chem. Sect. C.

[2] Rosen M.J., Kunjappu J.T. (2012). Surfactants and Interfacial Phenomena.

[3] Schwuger M.-J., Stickdorn K., Schomaecker R. (1995). Microemulsions in Technical Processes. Chem. Rev..

[4] Dow Chemical Corporate.

[5] Hirasaki G.J., Miller C.A., Puerto M. (2011). Recent Advances in Surfactant EOR. SPE J..

[6] Martínez-Palou R., Mosqueira M.L., Zapata-Rendón B., Mar-Juárez E., Bernal-Huicochea C., Clavel-López J.C., Aburto J. (2011). Transportation of heavy and extra-heavy crude oil by pipeline: A review. J. Petrol. Sci. Tech..

[7] Creton B., Nieto-Draghi C., Pannacci N. (2012). Prediction of Surfactants’ Properties using Multiscale Molecular Modeling Tools: A Review. Oil Gas Sci. Tech..

[8] Shelley J., Shelley M. (2000). Computer simulation of surfactant solutions. Curr. Opin. Colloid Interface Sci..

[9] Hernández-Trujillo J., García-Cruz I., Martínez-Magadán J.M. (2007). Molecular Characterization of p-Alkyl Phenol-n-Heptane Interactions and Their Implication as Asphaltene Dispersants. Energy Fuels.

[10] Warren P.B. (1998). Dissipative particle dynamics. Curr. Opin. Colloid Interface Sci..

[11] Chen S., Guo C., Hu G.-H., Liu H.-Z., Liang X.-F., Wang J., Ma J.-H., Zheng L. (2007). Dissipative particle dynamics simulation of gold nanoparticles stabilization by PEO–PPO–PEO block copolymer micelles. Colloid Polym. Sci..

[12] Groot R.D. (2000). Mesoscopic Simulation of Polymer-Surfactant Aggregation. Langmuir.

[13] Wu H., Xu J., He X., Zhao Y., Wen H. (2006). Mesoscopic simulation of self-assembly in surfactant oligomers by dissipative particle dynamics. Colloid Surf. A: Physicochem. Eng. Aspects.

[14] Barone V., Cossi M. (1998). Quantum Calculation of Molecular Energies and Energy Gradients in Solution by a Conductor Solvent Model. J. Phys. Chem. A.

[15] Os N.M. van, Haak J.R., Rupert L.A.M. (1993). Physico-Chemical Properties of Selected Anionic, Cationic and Noninonic Surfactants.

[16] Lam Y.-M., Goldbeck-Wood G. (2003). Mesoscale simulation of block copolymers in aqueous solution: parameterisation, micelle growth kinetics and the effect of temperature and concentration morphology. Polymer.

[17] Becke A.D. (1993). Functional thermochemistry. III. The role of exact exchange. J. Chem. Phys..

[18] Lee C., Yang W., Parr R.G. (1998). Development of the Colle–Salvetti correlation-energy formula into a functional of the electron density. Phys. Rev. B.

[19] Frisch M.J., Trucks G.W., Schlegel H.B., Scuseria G.E., Robb M.A., Cheeseman J.R., Montgomery J.A., Vreven T., Kudin K.N., Burant J.C. (2004). Gaussian 03.

[20] Groot R.D., Madden T.J. (1998). Dynamic simulation of diblock copolymer microphase separation. J. Chem. Phys..

[21] Groot R.D., Warren P.B. (1997). Dissipative particle dynamics: Bridging the gap between atomistic and mesoscopic simulation. J. Chem. Phys..

[22] Fan C.F., Olafson B.D., Blanco M., Hsu S.L. (1992). Application of molecular simulation to derive phase diagrams of binary mixtures. Macromolecules.

[23] Accelrys, Materials Studio.

